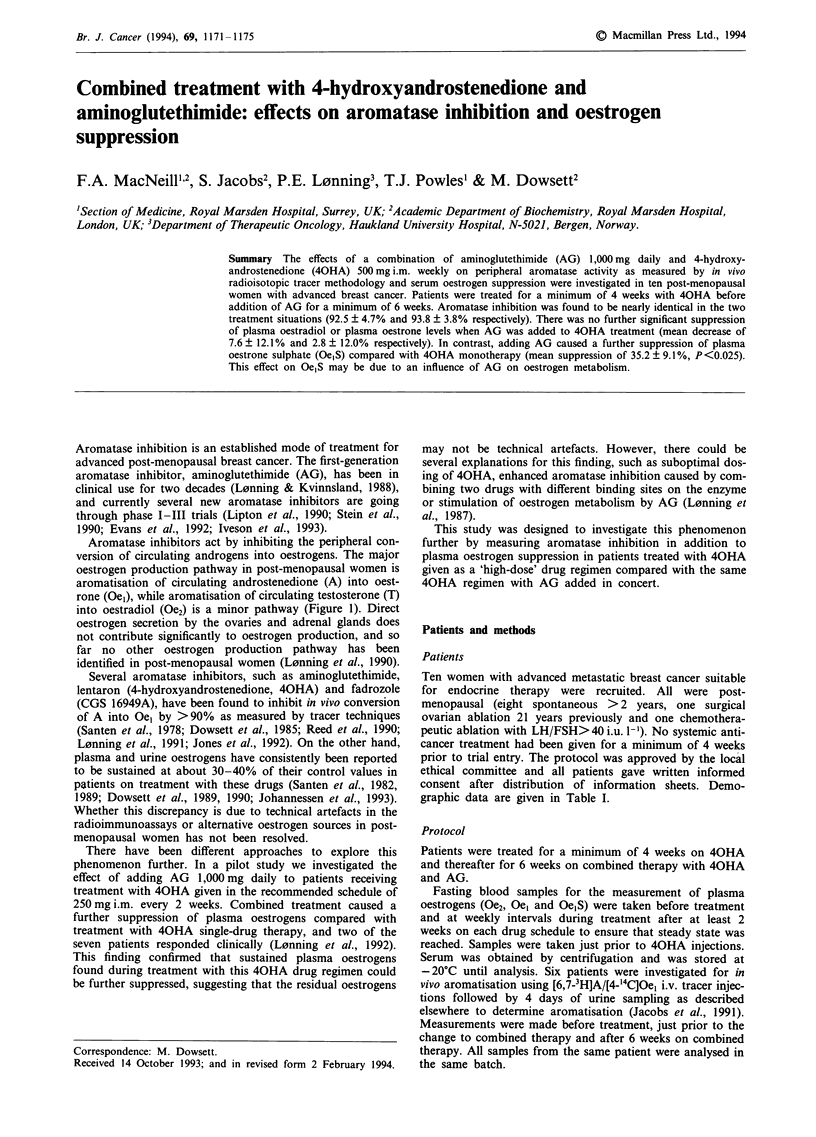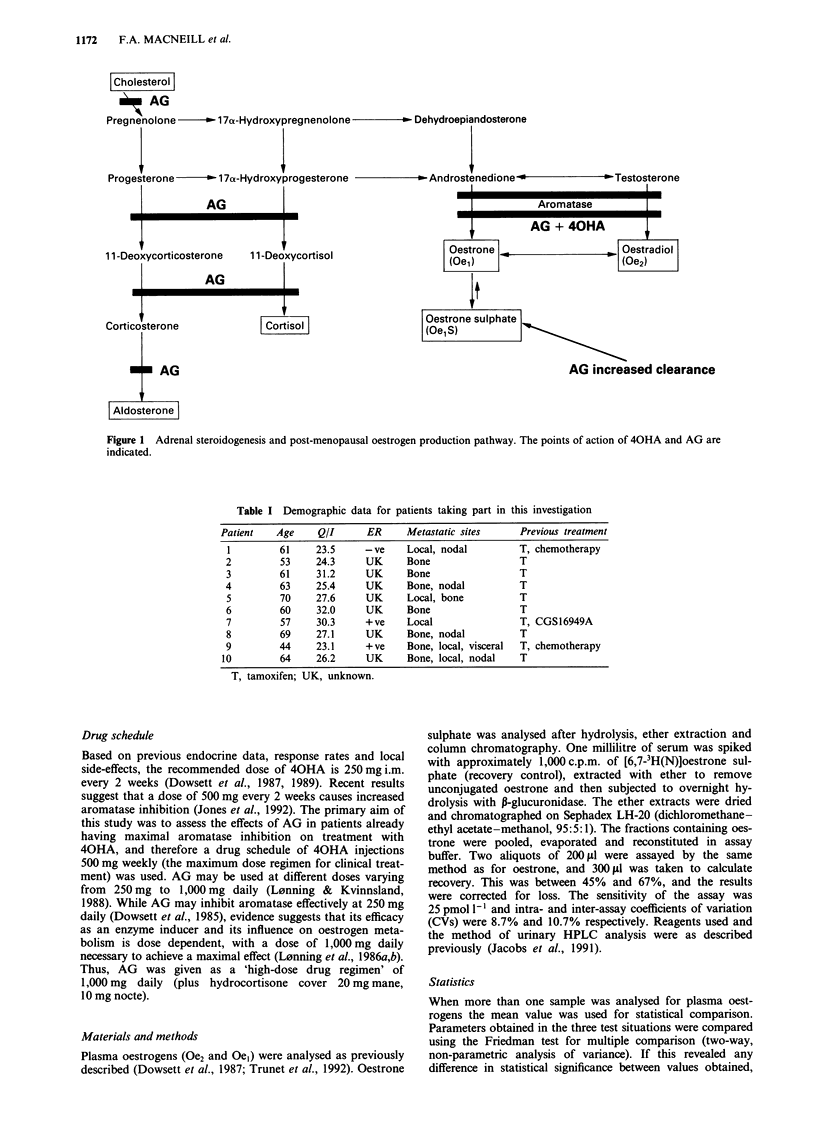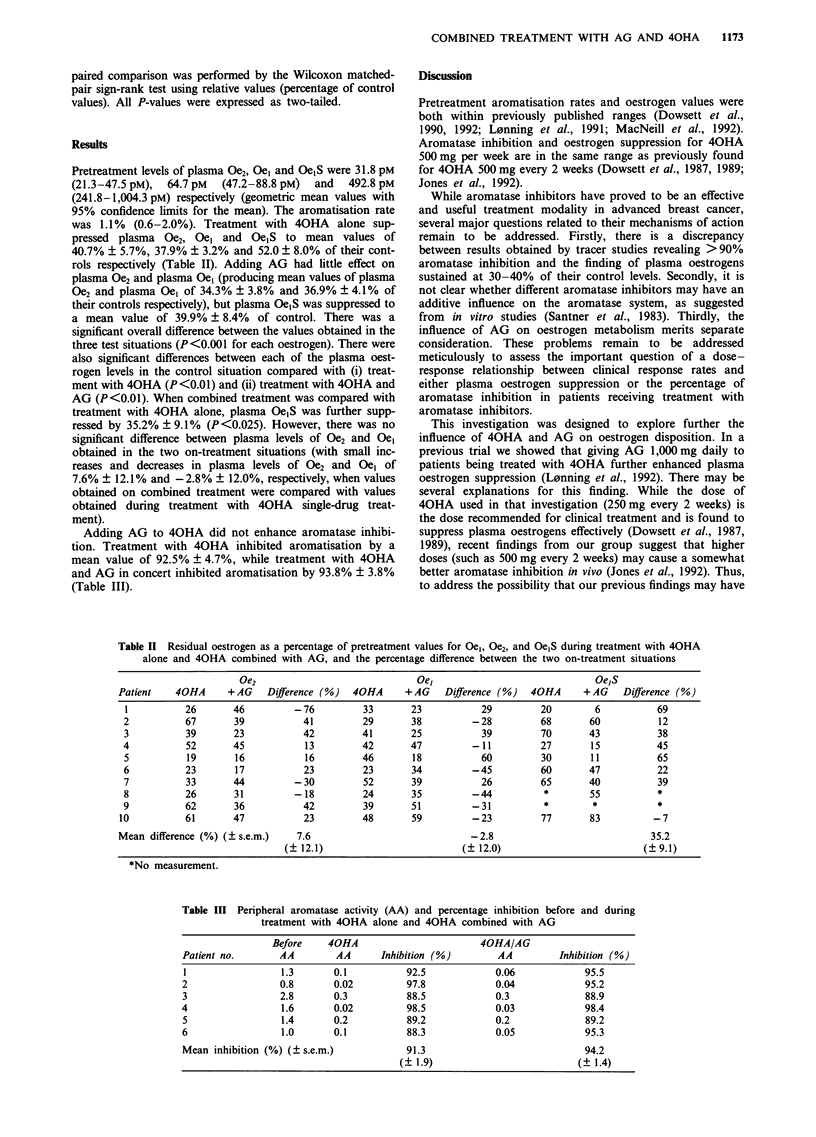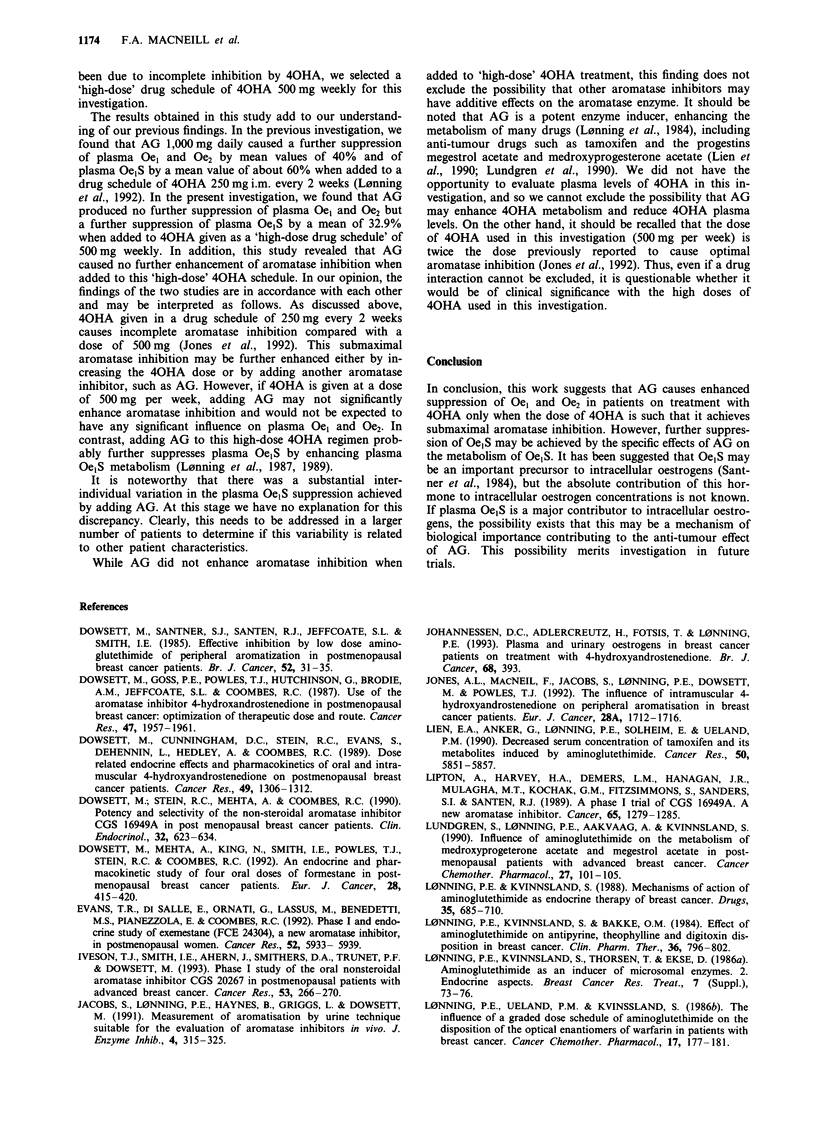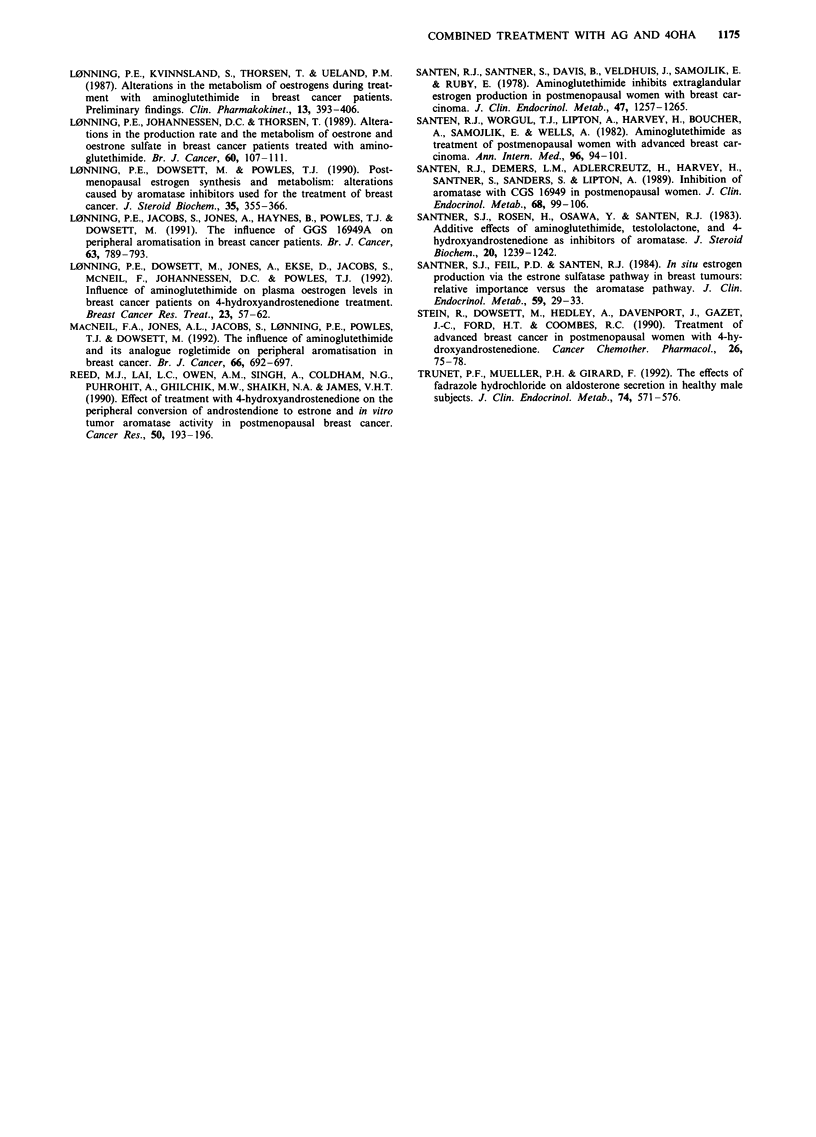# Combined treatment with 4-hydroxyandrostenedione and aminoglutethimide: effects on aromatase inhibition and oestrogen suppression.

**DOI:** 10.1038/bjc.1994.230

**Published:** 1994-06

**Authors:** F. A. MacNeill, S. Jacobs, P. E. Lønning, T. J. Powles, M. Dowsett

**Affiliations:** Section of Medicine, Royal Marsden Hospital, Surrey, UK.

## Abstract

The effects of a combination of aminoglutethimide (AG) 1,000 mg daily and 4-hydroxy-androstenedione (4OHA) 500 mg i.m. weekly on peripheral aromatase activity as measured by in vivo radioisotopic tracer methodology and serum oestrogen suppression were investigated in ten post-menopausal women with advanced breast cancer. Patients were treated for a minimum of 4 weeks with 4OHA before addition of AG for a minimum of 6 weeks. Aromatase inhibition was found to be nearly identical in the two treatment situations (92.5 +/- 4.7% and 93.8 +/- 3.8% respectively). There was no further significant suppression of plasma oestradiol or plasma oestrone levels when AG was added to 4OHA treatment (mean decrease of 7.6 +/- 12.1% and 2.8 +/- 12.0% respectively). In contrast, adding AG caused a further suppression of plasma oestrone sulphate (Oe1S) compared with 4OHA monotherapy (mean suppression of 35.2 +/- 9.1%, P < 0.025). This effect on Oe1S may be due to an influence of AG on oestrogen metabolism.


Br. J. Cancer (1994), 69, 1171–1175 © Macmillan Press Ltd., 1994

### Combined treatment with 4-hydroxyandrostenedione and aminoglutethimide: effects on aromatase inhibition and oestrogen suppression

F.A. MacNeill[1,2], S. Jacobs[2], P.E. Lønning[3], T.J. Powles[1] & M. Dowsett[2]

[1]*Section of Medicine, Royal Marsden Hospital, Surrey, UK;* [2]*Academic Department of Biochemistry, Royal Marsden Hospital, London, UK;* [3]*Department of Therapeutic Oncology, Haukland University Hospital, N-5021, Bergen, Norway.*

**Summary** The effects of a combination of aminoglutethimide (AG) 1,000 mg daily and 4-hydroxyandrostenedione (4OHA) 500 mg i.m. weekly on peripheral aromatase activity as measured by *in vivo* radioisotopic tracer methodology and serum oestrogen suppression were investigated in ten post-menopausal women with advanced breast cancer. Patients were treated for a minimum of 4 weeks with 4OHA before addition of AG for a minimum of 6 weeks. Aromatase inhibition was found to be nearly identical in the two treatment situations (92.5 ± 4.7% and 93.8 ± 3.8% respectively). There was no further significant suppression of plasma oestradiol or plasma oestrone levels when AG was added to 4OHA treatment (mean decrease of 7.6 ± 12.1% and 2.8 ± 12.0% respectively). In contrast, adding AG caused a further suppression of plasma oestrone sulphate ($Oe_1S$) compared with 4OHA monotherapy (mean suppression of 35.2 ± 9.1%, $P<0.025$). This effect on $Oe_1S$ may be due to an influence of AG on oestrogen metabolism.

Aromatase inhibition is an established mode of treatment for advanced post-menopausal breast cancer. The first-generation aromatase inhibitor, aminoglutethimide (AG), has been in clinical use for two decades (Lønning & Kvinnsland, 1988), and currently several new aromatase inhibitors are going through phase I–III trials (Lipton *et al.*, 1990; Stein *et al.*, 1990; Evans *et al.*, 1992; Iveson *et al.*, 1993).

Aromatase inhibitors act by inhibiting the peripheral conversion of circulating androgens into oestrogens. The major oestrogen production pathway in post-menopausal women is aromatisation of circulating androstenedione (A) into oestrone ($Oe_1$), while aromatisation of circulating testosterone (T) into oestradiol ($Oe_2$) is a minor pathway (Figure 1). Direct oestrogen secretion by the ovaries and adrenal glands does not contribute significantly to oestrogen production, and so far no other oestrogen production pathway has been identified in post-menopausal women (Lønning *et al.*, 1990).

Several aromatase inhibitors, such as aminoglutethimide, lentaron (4-hydroxyandrostenedione, 4OHA) and fadrozole (CGS 16949A), have been found to inhibit *in vivo* conversion of A into $Oe_1$ by >90% as measured by tracer techniques (Santen *et al.*, 1978; Dowsett *et al.*, 1985; Reed *et al.*, 1990; Lønning *et al.*, 1991; Jones *et al.*, 1992). On the other hand, plasma and urine oestrogens have consistently been reported to be sustained at about 30–40% of their control values in patients on treatment with these drugs (Santen *et al.*, 1982, 1989; Dowsett *et al.*, 1989, 1990; Johannessen *et al.*, 1993). Whether this discrepancy is due to technical artefacts in the radioimmunoassays or alternative oestrogen sources in post-menopausal women has not been resolved.

There have been different approaches to explore this phenomenon further. In a pilot study we investigated the effect of adding AG 1,000 mg daily to patients receiving treatment with 4OHA given in the recommended schedule of 250 mg i.m. every 2 weeks. Combined treatment caused a further suppression of plasma oestrogens compared with treatment with 4OHA single-drug therapy, and two of the seven patients responded clinically (Lønning *et al.*, 1992). This finding confirmed that sustained plasma oestrogens found during treatment with this 4OHA drug regimen could be further suppressed, suggesting that the residual oestrogens may not be technical artefacts. However, there could be several explanations for this finding, such as suboptimal dosing of 4OHA, enhanced aromatase inhibition caused by combining two drugs with different binding sites on the enzyme or stimulation of oestrogen metabolism by AG (Lønning *et al.*, 1987).

This study was designed to investigate this phenomenon further by measuring aromatase inhibition in addition to plasma oestrogen suppression in patients treated with 4OHA given as a 'high-dose' drug regimen compared with the same 4OHA regimen with AG added in concert.

### Patients and methods

### *Patients*

Ten women with advanced metastatic breast cancer suitable for endocrine therapy were recruited. All were post-menopausal (eight spontaneous >2 years, one surgical ovarian ablation 21 years previously and one chemotherapeutic ablation with LH/FSH>40 i.u. $l^{-1}$). No systemic anticancer treatment had been given for a minimum of 4 weeks prior to trial entry. The protocol was approved by the local ethical committee and all patients gave written informed consent after distribution of information sheets. Demographic data are given in Table I.

### *Protocol*

Patients were treated for a minimum of 4 weeks on 4OHA and thereafter for 6 weeks on combined therapy with 4OHA and AG.

Fasting blood samples for the measurement of plasma oestrogens ($Oe_2$, $Oe_1$ and $Oe_1S$) were taken before treatment and at weekly intervals during treatment after at least 2 weeks on each drug schedule to ensure that steady state was reached. Samples were taken just prior to 4OHA injections. Serum was obtained by centrifugation and was stored at −20°C until analysis. Six patients were investigated for *in vivo* aromatisation using $[6,7\text{-}^3H]A/[4\text{-}^{14}C]Oe_1$ i.v. tracer injections followed by 4 days of urine sampling as described elsewhere to determine aromatisation (Jacobs *et al.*, 1991). Measurements were made before treatment, just prior to the change to combined therapy and after 6 weeks on combined therapy. All samples from the same patient were analysed in the same batch.

Correspondence: M. Dowsett.
Received 14 October 1993; and in revised form 2 February 1994.

**Figure 1** Adrenal steroidogenesis and post-menopausal oestrogen production pathway. The points of action of 4OHA and AG are indicated.

**Table I** Demographic data for patients taking part in this investigation

| *Patient* | *Age* | *Q/I* | *ER* | *Metastatic sites* | *Previous treatment* |
|---|---|---|---|---|---|
| 1 | 61 | 23.5 | −ve | Local, nodal | T, chemotherapy |
| 2 | 53 | 24.3 | UK | Bone | T |
| 3 | 61 | 31.2 | UK | Bone | T |
| 4 | 63 | 25.4 | UK | Bone, nodal | T |
| 5 | 70 | 27.6 | UK | Local, bone | T |
| 6 | 60 | 32.0 | UK | Bone | T |
| 7 | 57 | 30.3 | +ve | Local | T, CGS16949A |
| 8 | 69 | 27.1 | UK | Bone, nodal | T |
| 9 | 44 | 23.1 | +ve | Bone, local, visceral | T, chemotherapy |
| 10 | 64 | 26.2 | UK | Bone, local, nodal | T |

T, tamoxifen; UK, unknown.

### *Drug schedule*

Based on previous endocrine data, response rates and local side-effects, the recommended dose of 4OHA is 250 mg i.m. every 2 weeks (Dowsett *et al.*, 1987, 1989). Recent results suggest that a dose of 500 mg every 2 weeks causes increased aromatase inhibition (Jones *et al.*, 1992). The primary aim of this study was to assess the effects of AG in patients already having maximal aromatase inhibition on treatment with 4OHA, and therefore a drug schedule of 4OHA injections 500 mg weekly (the maximum dose regimen for clinical treatment) was used. AG may be used at different doses varying from 250 mg to 1,000 mg daily (Lønning & Kvinnsland, 1988). While AG may inhibit aromatase effectively at 250 mg daily (Dowsett *et al.*, 1985), evidence suggests that its efficacy as an enzyme inducer and its influence on oestrogen metabolism is dose dependent, with a dose of 1,000 mg daily necessary to achieve a maximal effect (Lønning *et al.*, 1986*a*,*b*). Thus, AG was given as a 'high-dose drug regimen' of 1,000 mg daily (plus hydrocortisone cover 20 mg mane, 10 mg nocte).

### *Materials and methods*

Plasma oestrogens ($Oe_2$ and $Oe_1$) were analysed as previously described (Dowsett *et al.*, 1987; Trunet *et al.*, 1992). Oestrone sulphate was analysed after hydrolysis, ether extraction and column chromatography. One millilitre of serum was spiked with approximately 1,000 c.p.m. of [6,7-$^3$H(N)]oestrone sulphate (recovery control), extracted with ether to remove unconjugated oestrone and then subjected to overnight hydrolysis with β-glucuronidase. The ether extracts were dried and chromatographed on Sephadex LH-20 (dichloromethane–ethyl acetate–methanol, 95:5:1). The fractions containing oestrone were pooled, evaporated and reconstituted in assay buffer. Two aliquots of 200 μl were assayed by the same method as for oestrone, and 300 μl was taken to calculate recovery. This was between 45% and 67%, and the results were corrected for loss. The sensitivity of the assay was 25 pmol $l^{-1}$ and intra- and inter-assay coefficients of variation (CVs) were 8.7% and 10.7% respectively. Reagents used and the method of urinary HPLC analysis were as described previously (Jacobs *et al.*, 1991).

### *Statistics*

When more than one sample was analysed for plasma oestrogens the mean value was used for statistical comparison. Parameters obtained in the three test situations were compared using the Friedman test for multiple comparison (two-way, non-parametric analysis of variance). If this revealed any difference in statistical significance between values obtained, paired comparison was performed by the Wilcoxon matched-pair sign-rank test using relative values (percentage of control values). All $P$-values were expressed as two-tailed.

### Results

Pretreatment levels of plasma $Oe_2$, $Oe_1$ and $Oe_1S$ were 31.8 pM (21.3–47.5 pM), 64.7 pM (47.2–88.8 pM) and 492.8 pM (241.8–1,004.3 pM) respectively (geometric mean values with 95% confidence limits for the mean). The aromatisation rate was 1.1% (0.6–2.0%). Treatment with 4OHA alone suppressed plasma $Oe_2$, $Oe_1$ and $Oe_1S$ to mean values of 40.7% ± 5.7%, 37.9% ± 3.2% and 52.0 ± 8.0% of their controls respectively (Table II). Adding AG had little effect on plasma $Oe_2$ and plasma $Oe_1$ (producing mean values of plasma $Oe_2$ and plasma $Oe_1$ of 34.3% ± 3.8% and 36.9% ± 4.1% of their controls respectively), but plasma $Oe_1S$ was suppressed to a mean value of 39.9% ± 8.4% of control. There was a significant overall difference between the values obtained in the three test situations ($P<0.001$ for each oestrogen). There were also significant differences between each of the plasma oestrogen levels in the control situation compared with (i) treatment with 4OHA ($P<0.01$) and (ii) treatment with 4OHA and AG ($P<0.01$). When combined treatment was compared with treatment with 4OHA alone, plasma $Oe_1S$ was further suppressed by 35.2% ± 9.1% ($P<0.025$). However, there was no significant difference between plasma levels of $Oe_2$ and $Oe_1$ obtained in the two on-treatment situations (with small increases and decreases in plasma levels of $Oe_2$ and $Oe_1$ of 7.6% ± 12.1% and −2.8% ± 12.0%, respectively, when values obtained on combined treatment were compared with values obtained during treatment with 4OHA single-drug treatment).

Adding AG to 4OHA did not enhance aromatase inhibition. Treatment with 4OHA inhibited aromatisation by a mean value of 92.5% ± 4.7%, while treatment with 4OHA and AG in concert inhibited aromatisation by 93.8% ± 3.8% (Table III).

### Discussion

Pretreatment aromatisation rates and oestrogen values were both within previously published ranges (Dowsett *et al.*, 1990, 1992; Lønning *et al.*, 1991; MacNeill *et al.*, 1992). Aromatase inhibition and oestrogen suppression for 4OHA 500 mg per week are in the same range as previously found for 4OHA 500 mg every 2 weeks (Dowsett *et al.*, 1987, 1989; Jones *et al.*, 1992).

While aromatase inhibitors have proved to be an effective and useful treatment modality in advanced breast cancer, several major questions related to their mechanisms of action remain to be addressed. Firstly, there is a discrepancy between results obtained by tracer studies revealing >90% aromatase inhibition and the finding of plasma oestrogens sustained at 30–40% of their control levels. Secondly, it is not clear whether different aromatase inhibitors may have an additive influence on the aromatase system, as suggested from *in vitro* studies (Santner *et al.*, 1983). Thirdly, the influence of AG on oestrogen metabolism merits separate consideration. These problems remain to be addressed meticulously to assess the important question of a dose–response relationship between clinical response rates and either plasma oestrogen suppression or the percentage of aromatase inhibition in patients receiving treatment with aromatase inhibitors.

This investigation was designed to explore further the influence of 4OHA and AG on oestrogen disposition. In a previous trial we showed that giving AG 1,000 mg daily to patients being treated with 4OHA further enhanced plasma oestrogen suppression (Lønning *et al.*, 1992). There may be several explanations for this finding. While the dose of 4OHA used in that investigation (250 mg every 2 weeks) is the dose recommended for clinical treatment and is found to suppress plasma oestrogens effectively (Dowsett *et al.*, 1987, 1989), recent findings from our group suggest that higher doses (such as 500 mg every 2 weeks) may cause a somewhat better aromatase inhibition *in vivo* (Jones *et al.*, 1992). Thus, to address the possibility that our previous findings may have

**Table II** Residual oestrogen as a percentage of pretreatment values for $Oe_1$, $Oe_2$, and $Oe_1S$ during treatment with 4OHA alone and 4OHA combined with AG, and the percentage difference between the two on-treatment situations

| | $Oe_2$ | | | $Oe_1$ | | | $Oe_1S$ | | |
|---|---|---|---|---|---|---|---|---|---|
| *Patient* | *4OHA* | *+AG* | *Difference (%)* | *4OHA* | *+AG* | *Difference (%)* | *4OHA* | *+AG* | *Difference (%)* |
| 1 | 26 | 46 | −76 | 33 | 23 | 29 | 20 | 6 | 69 |
| 2 | 67 | 39 | 41 | 29 | 38 | −28 | 68 | 60 | 12 |
| 3 | 39 | 23 | 42 | 41 | 25 | 39 | 70 | 43 | 38 |
| 4 | 52 | 45 | 13 | 42 | 47 | −11 | 27 | 15 | 45 |
| 5 | 19 | 16 | 16 | 46 | 18 | 60 | 30 | 11 | 65 |
| 6 | 23 | 17 | 23 | 23 | 34 | −45 | 60 | 47 | 22 |
| 7 | 33 | 44 | −30 | 52 | 39 | 26 | 65 | 40 | 39 |
| 8 | 26 | 31 | −18 | 24 | 35 | −44 | * | 55 | * |
| 9 | 62 | 36 | 42 | 39 | 51 | −31 | * | * | * |
| 10 | 61 | 47 | 23 | 48 | 59 | −23 | 77 | 83 | −7 |
| Mean difference (%) (± s.e.m.) | | | 7.6 (± 12.1) | | | −2.8 (± 12.0) | | | 35.2 (± 9.1) |

*No measurement.

**Table III** Peripheral aromatase activity (AA) and percentage inhibition before and during treatment with 4OHA alone and 4OHA combined with AG

| *Patient no.* | *Before AA* | *4OHA AA* | *Inhibition (%)* | *4OHA/AG AA* | *Inhibition (%)* |
|---|---|---|---|---|---|
| 1 | 1.3 | 0.1 | 92.5 | 0.06 | 95.5 |
| 2 | 0.8 | 0.02 | 97.8 | 0.04 | 95.2 |
| 3 | 2.8 | 0.3 | 88.5 | 0.3 | 88.9 |
| 4 | 1.6 | 0.02 | 98.5 | 0.03 | 98.4 |
| 5 | 1.4 | 0.2 | 89.2 | 0.2 | 89.2 |
| 6 | 1.0 | 0.1 | 88.3 | 0.05 | 95.3 |
| Mean inhibition (%) (± s.e.m.) | | | 91.3 (± 1.9) | | 94.2 (± 1.4) |

been due to incomplete inhibition by 4OHA, we selected a 'high-dose' drug schedule of 4OHA 500 mg weekly for this investigation.

The results obtained in this study add to our understanding of our previous findings. In the previous investigation, we found that AG 1,000 mg daily caused a further suppression of plasma $Oe_1$ and $Oe_2$ by mean values of 40% and of plasma $Oe_1S$ by a mean value of about 60% when added to a drug schedule of 4OHA 250 mg i.m. every 2 weeks (Lønning *et al.*, 1992). In the present investigation, we found that AG produced no further suppression of plasma $Oe_1$ and $Oe_2$ but a further suppression of plasma $Oe_1S$ by a mean of 32.9% when added to 4OHA given as a 'high-dose drug schedule' of 500 mg weekly. In addition, this study revealed that AG caused no further enhancement of aromatase inhibition when added to this 'high-dose' 4OHA schedule. In our opinion, the findings of the two studies are in accordance with each other and may be interpreted as follows. As discussed above, 4OHA given in a drug schedule of 250 mg every 2 weeks causes incomplete aromatase inhibition compared with a dose of 500 mg (Jones *et al.*, 1992). This submaximal aromatase inhibition may be further enhanced either by increasing the 4OHA dose or by adding another aromatase inhibitor, such as AG. However, if 4OHA is given at a dose of 500 mg per week, adding AG may not significantly enhance aromatase inhibition and would not be expected to have any significant influence on plasma $Oe_1$ and $Oe_2$. In contrast, adding AG to this high-dose 4OHA regimen probably further suppresses plasma $Oe_1S$ by enhancing plasma $Oe_1S$ metabolism (Lønning *et al.*, 1987, 1989).

It is noteworthy that there was a substantial interindividual variation in the plasma $Oe_1S$ suppression achieved by adding AG. At this stage we have no explanation for this discrepancy. Clearly, this needs to be addressed in a larger number of patients to determine if this variability is related to other patient characteristics.

While AG did not enhance aromatase inhibition when added to 'high-dose' 4OHA treatment, this finding does not exclude the possibility that other aromatase inhibitors may have additive effects on the aromatase enzyme. It should be noted that AG is a potent enzyme inducer, enhancing the metabolism of many drugs (Lønning *et al.*, 1984), including anti-tumour drugs such as tamoxifen and the progestins megestrol acetate and medroxyprogesterone acetate (Lien *et al.*, 1990; Lundgren *et al.*, 1990). We did not have the opportunity to evaluate plasma levels of 4OHA in this investigation, and so we cannot exclude the possibility that AG may enhance 4OHA metabolism and reduce 4OHA plasma levels. On the other hand, it should be recalled that the dose of 4OHA used in this investigation (500 mg per week) is twice the dose previously reported to cause optimal aromatase inhibition (Jones *et al.*, 1992). Thus, even if a drug interaction cannot be excluded, it is questionable whether it would be of clinical significance with the high doses of 4OHA used in this investigation.

### Conclusion

In conclusion, this work suggests that AG causes enhanced suppression of $Oe_1$ and $Oe_2$ in patients on treatment with 4OHA only when the dose of 4OHA is such that it achieves submaximal aromatase inhibition. However, further suppression of $Oe_1S$ may be achieved by the specific effects of AG on the metabolism of $Oe_1S$. It has been suggested that $Oe_1S$ may be an important precursor to intracellular oestrogens (Santner *et al.*, 1984), but the absolute contribution of this hormone to intracellular oestrogen concentrations is not known. If plasma $Oe_1S$ is a major contributor to intracellular oestrogens, the possibility exists that this may be a mechanism of biological importance contributing to the anti-tumour effect of AG. This possibility merits investigation in future trials.

### References

DOWSETT, M., SANTNER, S.J., SANTEN, R.J., JEFFCOATE, S.L. & SMITH, I.E. (1985). Effective inhibition by low dose aminoglutethimide of peripheral aromatization in postmenopausal breast cancer patients. *Br. J. Cancer*, **52**, 31–35.

DOWSETT, M., GOSS, P.E., POWLES, T.J., HUTCHINSON, G., BRODIE, A.M., JEFFCOATE, S.L. & COOMBES, R.C. (1987). Use of the aromatase inhibitor 4-hydroxandrostenedione in postmenopausal breast cancer: optimization of therapeutic dose and route. *Cancer Res.*, **47**, 1957–1961.

DOWSETT, M., CUNNINGHAM, D.C., STEIN, R.C., EVANS, S., DEHENNIN, L., HEDLEY, A. & COOMBES, R.C. (1989). Dose related endocrine effects and pharmacokinetics of oral and intramuscular 4-hydroxyandrostenedione on postmenopausal breast cancer patients. *Cancer Res.*, **49**, 1306–1312.

DOWSETT, M.; STEIN, R.C., MEHTA, A. & COOMBES, R.C. (1990). Potency and selectivity of the non-steroidal aromatase inhibitor CGS 16949A in post menopausal breast cancer patients. *Clin. Endocrinol.*, **32**, 623–634.

DOWSETT, M., MEHTA, A., KING, N., SMITH, I.E., POWLES, T.J., STEIN, R.C. & COOMBES, R.C. (1992). An endocrine and pharmacokinetic study of four oral doses of formestane in postmenopausal breast cancer patients. *Eur. J. Cancer*, **28**, 415–420.

EVANS, T.R., DI SALLE, E., ORNATI, G., LASSUS, M., BENEDETTI, M.S., PIANEZZOLA, E. & COOMBES, R.C. (1992). Phase I and endocrine study of exemestane (FCE 24304), a new aromatase inhibitor, in postmenopausal women. *Cancer Res.*, **52**, 5933– 5939.

IVESON, T.J., SMITH, I.E., AHERN, J., SMITHERS, D.A., TRUNET, P.F. & DOWSETT, M. (1993). Phase I study of the oral nonsteroidal aromatase inhibitor CGS 20267 in postmenopausal patients with advanced breast cancer. *Cancer Res.*, **53**, 266–270.

JACOBS, S., LØNNING, P.E., HAYNES, B., GRIGGS, L. & DOWSETT, M. (1991). Measurement of aromatisation by urine technique suitable for the evaluation of aromatase inhibitors *in vivo*. *J. Enzyme Inhib.*, **4**, 315–325.

JOHANNESSEN, D.C., ADLERCREUTZ, H., FOTSIS, T. & LØNNING, P.E. (1993). Plasma and urinary oestrogens in breast cancer patients on treatment with 4-hydroxyandrostenedione. *Br. J. Cancer*, **68**, 393.

JONES, A.L., MACNEIL, F., JACOBS, S., LØNNING, P.E., DOWSETT, M. & POWLES, T.J. (1992). The influence of intramuscular 4-hydroxyandrostenedione on peripheral aromatisation in breast cancer patients. *Eur. J. Cancer*, **28A**, 1712–1716.

LIEN, E.A., ANKER, G., LØNNING, P.E., SOLHEIM, E. & UELAND, P.M. (1990). Decreased serum concentration of tamoxifen and its metabolites induced by aminoglutethimide. *Cancer Res.*, **50**, 5851–5857.

LIPTON, A., HARVEY, H.A., DEMERS, L.M., HANAGAN, J.R., MULAGHA, M.T., KOCHAK, G.M., FITZSIMMONS, S., SANDERS, S.I. & SANTEN, R.J. (1989). A phase I trial of CGS 16949A. A new aromatase inhibitor. *Cancer*, **65**, 1279–1285.

LUNDGREN, S., LØNNING, P.E., AAKVAAG, A. & KVINNSLAND, S. (1990). Influence of aminoglutethimide on the metabolism of medroxyprogeterone acetate and megestrol acetate in postmenopausal patients with advanced breast cancer. *Cancer Chemother. Pharmacol.*, **27**, 101–105.

LØNNING, P.E. & KVINNSLAND, S. (1988). Mechanisms of action of aminoglutethimide as endocrine therapy of breast cancer. *Drugs*, **35**, 685–710.

LØNNING, P.E., KVINNSLAND, S. & BAKKE, O.M. (1984). Effect of aminoglutethimide on antipyrine, theophylline and digitoxin disposition in breast cancer. *Clin. Pharm. Ther.*, **36**, 796–802.

LØNNING, P.E., KVINNSLAND, S., THORSEN, T. & EKSE, D. (1986*a*). Aminoglutethimide as an inducer of microsomal enzymes. 2. Endocrine aspects. *Breast Cancer Res. Treat.*, **7** (Suppl.), 73–76.

LØNNING, P.E., UELAND, P.M. & KVINSSLAND, S. (1986*b*). The influence of a graded dose schedule of aminoglutethimide on the disposition of the optical enantiomers of warfarin in patients with breast cancer. *Cancer Chemother. Pharmacol.*, **17**, 177–181.

LØNNING, P.E., KVINNSLAND, S., THORSEN, T. & UELAND, P.M. (1987). Alterations in the metabolism of oestrogens during treatment with aminoglutethimide in breast cancer patients. Preliminary findings. *Clin. Pharmakokinet.*, **13**, 393–406.

LØNNING, P.E., JOHANNESSEN, D.C. & THORSEN, T. (1989). Alterations in the production rate and the metabolism of oestrone and oestrone sulfate in breast cancer patients treated with aminoglutethimide. *Br. J. Cancer*, **60**, 107–111.

LØNNING, P.E., DOWSETT, M. & POWLES, T.J. (1990). Postmenopausal estrogen synthesis and metabolism: alterations caused by aromatase inhibitors used for the treatment of breast cancer. *J. Steroid Biochem.*, **35**, 355–366.

LØNNING, P.E., JACOBS, S., JONES, A., HAYNES, B., POWLES, T.J. & DOWSETT, M. (1991). The influence of GGS 16949A on peripheral aromatisation in breast cancer patients. *Br. J. Cancer*, **63**, 789–793.

LØNNING, P.E., DOWSETT, M., JONES, A., EKSE, D., JACOBS, S., MCNEIL, F., JOHANNESSEN, D.C. & POWLES, T.J. (1992). Influence of aminoglutethimide on plasma oestrogen levels in breast cancer patients on 4-hydroxyandrostenedione treatment. *Breast Cancer Res. Treat.*, **23**, 57–62.

MACNEIL, F.A., JONES, A.L., JACOBS, S., LØNNING, P.E., POWLES, T.J. & DOWSETT, M. (1992). The influence of aminoglutethimide and its analogue rogletimide on peripheral aromatisation in breast cancer. *Br. J. Cancer*, **66**, 692–697.

REED, M.J., LAI, L.C., OWEN, A.M., SINGH, A., COLDHAM, N.G., PUHROHIT, A., GHILCHIK, M.W., SHAIKH, N.A. & JAMES, V.H.T. (1990). Effect of treatment with 4-hydroxyandrostenedione on the peripheral conversion of androstendione to estrone and *in vitro* tumor aromatase activity in postmenopausal breast cancer. *Cancer Res.*, **50**, 193–196.

SANTEN, R.J., SANTNER, S., DAVIS, B., VELDHUIS, J., SAMOJLIK, E. & RUBY, E. (1978). Aminoglutethimide inhibits extraglandular estrogen production in postmenopausal women with breast carcinoma. *J. Clin. Endocrinol. Metab.*, **47**, 1257–1265.

SANTEN, R.J., WORGUL, T.J., LIPTON, A., HARVEY, H., BOUCHER, A., SAMOJLIK, E. & WELLS, A. (1982). Aminoglutethimide as treatment of postmenopausal women with advanced breast carcinoma. *Ann. Intern. Med.*, **96**, 94–101.

SANTEN, R.J., DEMERS, L.M., ADLERCREUTZ, H., HARVEY, H., SANTNER, S., SANDERS, S. & LIPTON, A. (1989). Inhibition of aromatase with CGS 16949 in postmenopausal women. *J. Clin. Endocrinol. Metab.*, **68**, 99–106.

SANTNER, S.J., ROSEN, H., OSAWA, Y. & SANTEN, R.J. (1983). Additive effects of aminoglutethimide, testololactone, and 4-hydroxyandrostenedione as inhibitors of aromatase. *J. Steroid Biochem.*, **20**, 1239–1242.

SANTNER, S.J., FEIL, P.D. & SANTEN, R.J. (1984). *In situ* estrogen production via the estrone sulfatase pathway in breast tumours: relative importance versus the aromatase pathway. *J. Clin. Endocrinol. Metab.*, **59**, 29–33.

STEIN, R., DOWSETT, M., HEDLEY, A., DAVENPORT, J., GAZET, J.-C., FORD, H.T. & COOMBES, R.C. (1990). Treatment of advanced breast cancer in postmenopausal women with 4-hydroxyandrostenedione. *Cancer Chemother. Pharmacol.*, **26**, 75–78.

TRUNET, P.F., MUELLER, P.H. & GIRARD, F. (1992). The effects of fadrazole hydrochloride on aldosterone secretion in healthy male subjects. *J. Clin. Endocrinol. Metab.*, **74**, 571–576.